# Management of Invasive Fungal Infections in Pediatric Acute Leukemia and the Appropriate Time for Restarting Chemotherapy

**DOI:** 10.4274/tjh.2014.0035

**Published:** 2015-12-03

**Authors:** Özlem Tüfekçi, Şebnem Yılmaz Bengoa, Fatma Demir Yenigürbüz, Erdem Şimşek, Tuba Hilkay Karapınar, Gülersu İrken, Hale Ören

**Affiliations:** 1 Dokuz Eylül University Faculty of Medicine, Department of Pediatric Hematology, İzmir, Turkey; 2 Dokuz Eylül University Faculty of Medicine, Department of Pediatrics, İzmir, Turkey

**Keywords:** Acute leukemia, Chemotherapy, children, fungal infection

## Abstract

**Objective:**

Rapid and effective treatment of invasive fungal infection (IFI) in patients with leukemia is important for survival. In this study, we aimed to describe variations regarding clinical features, treatment modalities, time of restarting chemotherapy, and outcome in children with IFI and acute leukemia (AL).

**Materials and Methods:**

The charts of all pediatric AL patients in our clinic between the years of 2001 and 2013 were retrospectively reviewed. All patients received prophylactic fluconazole during the chemotherapy period.

**Results:**

IFI was identified in 25 (14%) of 174 AL patients. Most of them were in the consolidation phase of chemotherapy and the patients had severe neutropenia. The median time between leukemia diagnosis and definition of IFI was 122 days. Twenty-four patients had pulmonary IFI. The most frequent finding on computed tomography was typical parenchymal nodules. The episodes were defined as proven in 4 (16%) patients, probable in 7 (28%) patients, and possible in 14 (56%) patients. The median time for discontinuation of chemotherapy was 27 days. IFI was treated successfully in all patients with voriconazole, amphotericin B, caspofungin, or posaconazole alone or in combination. Chemotherapy was restarted in 50% of the patients safely within 4 weeks and none of those patients experienced reactivation of IFI. All of them were given secondary prophylaxis. The median time for antifungal treatment and for secondary prophylaxis was 26 and 90 days, respectively. None of the patients died due to IFI.

**Conclusion:**

Our data show that rapid and effective antifungal therapy with rational treatment modalities may decrease the incidence of death and that restarting chemotherapy within several weeks may be safe in children with AL and IFI.

## Abstract (tr)

**Amaç:**

Lösemili hastalarda invaziv fungal enfeksiyonların (İFE) çabuk ve etkin tedavisi sağkalım için önemlidir. Bu çalışmada akut lösemi (AL) ve İFE olan çocuklarda klinik bulgular, tedavi şekilleri, tekrar kemoterapiye başlama zamanı ve tedavi sonucu gibi değişkenleri değerlendirmeyi amaçladık.

**Gereç ve Yöntemler:**

Kliniğimizde 2001-2013 yılları arasında izlenmiş tüm AL’lı çocukların hastane kayıtları retrospektif olarak tarandı. Tüm hastalara kemoterapi süresince proflaktik flukonazol tedavisi verildi.

**Bulgular:**

İFE, 174 AL hastasından 25’inde (%14) saptandı. Çoğu konsolidasyon tedavisi sırasında gelişmişti ve hastalar ağır nötropenikti. Lösemi tanısı ve İFE gelişme arasındaki ortanca süre 122 gündü. Hastaların 24’ünde pulmoner İFE vardı. Bilgisayarlı tomografi tetkikinde en sık izlenen bulgu parenkimal nodüllerdi. İFE epizodları 4 (%16) olguda kanıtlanmış, 7 (%28) olguda olası, 14 (%56) olguda muhtemel olarak değerlendirildi. Kemoterapiye ara verme süresi ortanca 27 gündü. İFE voriconazole, amphotericin B, caspofungin, posaconazole tekli veya kombine tedavileri ile başarıyla tedavi edildi. Olguların %50’sinde kemoterapiye 4 haftadan önce başlandı ve hiçbirinde İFE reaktivasyonu saptanmadı. Tümüne ikincil proflaksi verildi. Antifungal tedavi ve sekonder proflaksi ortanca süresi sırayla 26 ve 90 gündü. Hastalardan hiçbiri İFE ile kaybedilmedi.

**Sonuç:**

Verilerimiz AL ve İFE olan çocuklarda erken ve etkin rasyonel antifungal tedavi ile ölüm oranının azaltılabileceğini ve birkaç hafta içinde kemoterapiye güvenle başlanabileceğini göstermektedir.

## INTRODUCTION

The breakdown of host defense mechanisms in immunocompromised patients leads to increased risk of life-threatening infections, including invasive fungal infections (IFIs) [1,2,3,4,5,6]. Studies of pediatric populations with hemato-oncological diseases show an incidence rate of IFI ranging from 4.9% to 29% [7,8,9,10]. Besides causing increased mortality and morbidity, IFIs cause a substantial delay in treatment of acute leukemia (AL), which in turn could result in failure of this potentially curative treatment. The optimal time for restarting chemotherapy in these patients is not clear.

In this retrospective study, our purpose was to describe the incidence, risk factors, clinical features, treatment modalities, and outcome of IFIs in children with AL. We also aimed to investigate the appropriate (optimal) time for restarting chemotherapy in this group of patients.

## MATERIALS AND METHODS

### Patients and Institution

This retrospective study included all acute lymphoblastic leukemia (ALL) and acute myeloid leukemia (AML) patients, aged 0-18 years, who developed IFI at our clinic between January 2001 and January 2013. The patients were identified by reviewing the medical charts of all AL patients. Children with ALL received the BFM-95 or BFM-2000 protocol and those with AML received the BFM-98 or BFM-2004 protocol. Children were hospitalized in single rooms without high-efficiency air filtration systems.

The medical, microbiological, and imaging records of the patients who met the inclusion criteria were reviewed for the following variables:

### Demographic and clinical data:

Age and sex, leukemia type, remission status, and risk group of underlying disease at the time of diagnosis; the day and phase of treatment at which IFI developed (remission, induction, consolidation, maintenance); corticosteroid use 14 days prior to IFI onset; presence of central venous catheter; presence of mucositis; duration of neutropenia prior to IFI; use and type of primary antifungal prophylaxis; type of symptoms and signs of the IFI.

### Laboratory data:

Complete blood count; fungus detection tests including serum galactomannan (GM) antigen, direct stains, cultures, sinus aspirate, and samples from other sites.

### Radiological data:

X-ray, computed tomography, and ultrasound.

### Treatment and outcome of IFI:

Empiric therapy and definitive therapy with one or a combination of antifungal drugs; use of surgery; duration of treatment; the time from the onset of fungal infection to the restarting of chemotherapy; the use and type of secondary antifungal prophylaxis; the development of reactivation of fungal infection; mortality.

According to our institutional policy, all patients with ALL and AML receive prophylactic fluconazole (4-6 mg/kg/day) during all phases of chemotherapy. Severe neutropenia was defined as absolute granulocyte count of <500/mm3.

Serum GM assay could not be done routinely for all patients as the assay was not always available in the hospital laboratory. A positive result was based on 2 consecutive samples with a GM index of greater than or equal to 0.5.

Radiologic evaluation was performed after 5 days of fever, including high-resolution computed tomography (HRCT) of the chest and paranasal sinuses, in addition to cardiac echocardiogram.

The primary empiric antifungal treatment in our clinic was either with caspofungin or liposomal amphotericin B, depending on availability in the pharmacy of the hospital. The antifungal agent was sometimes switched during the course of the illness if the patient was intolerant or in culture-positive cases, according to the drug susceptibilities of the specific pathogen isolated.

In all cases, IFI was defined according to the guidelines of the EORTC/MSG [11]. Proven IFI was diagnosed by a positive fungal culture from a normally sterile site. Probable IFI was diagnosed on the basis of a combination of host factors, clinical and radiological features, and mycological evidence, such as positive fungal culture, positive GM assay, or microscopy of bronchoalveolar lavage fluid or sinus aspirate. Possible IFI was diagnosed when the clinical and imaging findings and host factors were consistent with IFI but there was no mycological support.

### Statistical Analysis

Statistical analyses were performed with SPSS 15. Descriptive statistics were calculated and reported as absolute frequencies or percentages for qualitative data and as medians and ranges for quantitative data.

## RESULTS

A total of 174 patients were diagnosed with and treated for AL (144 had ALL and 30 had AML) in our clinic. IFI was diagnosed in 25 (14%) of 174 AL patients, in 12% of all ALL cases, and in 27% of all AML cases. The characteristics of the 25 patients diagnosed with IFI are shown in Table 1. Of the 25 patients, 17 (68%) had ALL and 8 (32%) had AML. Five of the 8 AML patients (62%) and 7 of the 17 ALL patients (41%) were allocated into the high-risk group at the time of diagnosis of AL. The median age was 12 years (range: 0.7-17.5 years). Nine (36%) of the patients were in the induction phase, 14 (56%) of the patients were in the consolidation phase, and 2 (8%) of the patients were in the maintenance phase of chemotherapy; overall, 18 (72%) patients were in remission at the time of diagnosis of IFI.

The median time between the leukemia diagnosis and the definition of IFI was 122 days (range: 15-305 days). Absolute neutrophil count was <500/mm3 in 86% of patients. The median time for duration of neutropenia was 13 days (range: 0-47 days). All of the patients were febrile at the time of diagnosis. Of the 25 AL patients with IFI, 23 patients had isolated pulmonary IFI, 1 patient had isolated orbitocerebral aspergillosis infection, and 1 patient had both orbitocerebral and pulmonary Mucor and aspergillosis infection. The episodes of IFI were defined as proven in 4 (16%) patients, probable in 7 (28%) patients, and possible in 14 (56%) patients (Table 2).

Only 7 (29%) of the 24 patients with pulmonary IFI had positive pulmonary auscultatory findings. All of the patients with pulmonary IFI had positive findings on pulmonary HRCT, the most common being typical parenchymal nodules in 17 (70%) of 24 patients. Two patients had both halo signs and air-crescent findings on HRCT, while 1 patient had only halo signs. Air-crescent findings alone were present in 2 cases. The remaining 4 patients had consolidation areas on HRCT. Eleven (45%) of the 24 patients with pulmonary IFI had concomitant positive chest X-ray findings at the time of diagnosis. Of the 11 patients with positive chest X-ray findings, 9 patients had parenchymal infiltration, 2 patients had nodular infiltration, and 1 had pleural effusion. GM was screened in 21 patients and, of those, 9 (42%) had positive GM antigenemia.

IFI was treated successfully in all patients with voriconazole, amphotericin B, caspofungin, or posaconazole alone or in combination. The 2 patients with orbitocerebral IFI needed surgery. The initial treatment was voriconazole in 11 patients, liposomal amphotericin B in 7 patients, caspofungin in 4 patients, and conventional amphotericin B in 3 patients. The median time for total antifungal treatment was 26 days (range: 21-57 days). All patients were given secondary prophylaxis with oral voriconazole, itraconazole, or posaconazole. The median time for secondary prophylaxis was 90 days (range: 39-429 days). Reactivation of IFI occurred in 4 patients as pulmonary IFI; all of them were cured completely after treatment.

The median time for discontinuation of chemotherapy was 27 days (range: 0-57 days). Chemotherapy was not restarted in 3 patients due to refractory/progressive primary disease. Out of 22 patients for whom chemotherapy was restarted, the duration of cessation of chemotherapy was <14 days in 5 (23%) patients and 14-28 days in 6 (27%) patients (Table 3). Overall, chemotherapy was restarted in 50% of the patients safely before 4 weeks, and none of those patients experienced reactivation of IFI. For those for whom the chemotherapy was restarted after 28 days, the median time for discontinuation of chemotherapy was 35 days (range: 30-57 days). Regarding the outcome of the primary disease, the leukemia was cured in 9 of 11 patients for whom chemotherapy was started before 28 days and in 7 of 11 patients for whom the chemotherapy was started after that time. The median time for radiological improvement (no pathological signs in HRCT) was 20 days (range: 7-180 days) in the patients for whom chemotherapy was started after 28 days, while it was 17 days (range: 8-48 days) in those for whom chemotherapy was started before 28 days.

Death occurred in 9 (36%) patients (Table 4). Six of the patients who died were in the high-risk group.

## DISCUSSION

In the present retrospective study, IFI was defined in 14% of 174 children with AL. The incidence of IFI has been reported as between 1.3% and 25% in pediatric patients with hematological malignancies [4,7,12,13]. Taking into account the patient population and the conditions of the health center, our rate is very similar to the rate of another retrospective study from Turkey, which reported the incidence rate of IFI as 13.6% [10]. Another Turkish study reported the incidence rate of proven and probable IFI as 14.3% in patients with AML, ALL, and aplastic anemia [14].

Previous studies have suggested that older age is a risk factor for IFI in children [7,15,16]. The median age was 12 years in our study. Dvorak et al. and Kobayashi et al. found that age above 10 years on admission is a risk factor for IFI and it has been suggested that this finding may reflect the importance of host colonization by environmental fungi as an important step in the development of invasive disease, with younger patients having had less exposure time to fungal spores in the environment [15,16,17].

The majority of our patients (88%) were severely neutropenic at the time of diagnosis of IFI and the overall median duration of neutropenia was longer than 10 days. The incidence of IFI in children with leukemia was previously found to be closely related to the type of leukemia, with AML having a higher rate than ALL as in our study [3,7,18,19,20]. The intensive treatment and the relatively longer duration of neutropenia in AML patients are responsible for the increased risk of infections in this group of patients.

More than half of our patients (56%) were in the consolidation phase at the time of diagnosis of IFI. Similarly, Hale et al. also reported that half of IFIs were diagnosed 100-365 days after the initial diagnosis in AL patients [12]. We use BFM protocols and the consolidation phases of ALL and AML in BFM protocols correspond to HD-MTX and HD-ARA C blocks where there is increased risk of mucositis, a known risk factor for fungal infections [21,22].

In our study, GM was positive in 2 consecutive samples of 9 patients. Adult studies and recent pediatric studies have revealed the favorable specificity of the assay [23,24,25,26,27,28,29]. Another important diagnostic approach in identifying IFI is the HRCT of the chest. Chest X-rays have little value in the early stage of disease [30,31,32]. The most common sign in our patients was typical parenchymal nodules on HRCT; halo signs and air-crescent findings were less frequently seen. It is important to emphasize that pulmonary lesions characteristic for adults, such as air-crescent signs and cavitary lesions, are rarely seen in children [33,34]. A recent retrospective analysis of 139 pediatric invasive aspergillosis cases reported that the most frequent diagnostic radiologic finding was nodules at a rate of 34.6% [35].

The vast majority of IFIs in our study were due to Aspergillus spp. and the respiratory tract was the most common site for invasive aspergillosis. On the other hand, the absence of Candida albicans infections was remarkable in our study, which may be attributable to the strict use of fluconazole. One of our patients had Candida kefyr bloodstream infection, which is the fluconazole-resistant nonalbicans type of Candida and may be seen in patients with neutropenia. Recent reports have shown that infections caused by resistant Candida spp. and molds such as Aspergillus, Fusarium, and Scedosporium have been subsequently increased by the widespread use of fluconazole prophylaxis [10,12,36,37]. An additional risk factor for development of invasive aspergillosis in our study might be the absence of effective air filtration systems in patient rooms, as well as ongoing hospital renovation for the last 5 years. There are many reports in the literature suggesting an association between invasive aspergillosis and contaminated ventilation systems, hospital construction, or renovation [14,38,39].

Empirical antifungal therapy and investigation for IFIs should be considered for patients with persistent or recurrent fever after 4-7 days of antibiotics [40,41]. IFI was treated successfully in all our patients with voriconazole, amphotericin B, caspofungin, or posaconazole alone or in combination [42,43,44,45,46]. One of our patients with orbitocerebral mucormycosis and aspergillosis initially did not respond to liposomal amphotericin B, but did recover completely after posaconazole was added to the treatment. Combination therapy, although not recommended by international guidelines, is often used as rescue treatment in patients who are switched to second- or third-line antifungal therapy [45,47]. Regarding secondary antifungal prophylaxis, it is recommended to continue treatment with an agent and dose effective against the isolate of the primary infection until the end of immunosuppression [48].

An important consequence of IFI is that the relatively longer duration of time for treatment of this severe infection causes a significant delay in the primary treatment of AL. The optimal time for restarting chemotherapy in these patients is not clear, which poses a great dilemma for the physician [45]. One of our aims in this study was to investigate the safe, appropriate timing for restarting chemotherapy in these patients. The median time for discontinuation of chemotherapy was 27 days in our study; chemotherapy was restarted in 50% of the patients safely before 4 weeks and none of those patients experienced reactivation of IFI. Similarly Nosari et al., in their retrospective review of hematological malignancies, identified 61 adult cases of IFI and detected a median time of 27 days for discontinuation of chemotherapy (range: 17-45 days) [49]. The decision for timing chemotherapy is generally made on an individual basis depending on the extent of the fungal disease and the status of the primary disease.

The mortality rate of IFI shows wide variations among the studies reported in the literature. While earlier studies reported IFI-related mortality rates of up to 85%, recent studies have reported lower rates [8,39,50,51,52]. Kaya et al. reported the rate of IFI-attributable death as 5% (1 patient) in 21 children with AL [10]. Another previously mentioned study from Turkey found the total mortality of IFI to be 30% in 23 patients with AL and aplastic anemia [14]. In this study, death occurred in 36% of patients, but none of the deaths were attributable to the IFIs themselves. This finding may be due to increased awareness of the possibility of IFIs, the widespread use of HRCT as an early diagnostic method, early empirical treatment for febrile neutropenic patients, and greater effectiveness of newer antifungal agents.

In conclusion, our study demonstrated that rapid and effective antifungal therapy with rational treatment modalities may decrease the incidence of death in children with AL and IFI. Depending on the clinical status of the patient, restarting chemotherapy within several weeks may be safe and reactivation of IFI may be prevented with secondary prophylaxis.

## Figures and Tables

**Table 1:**
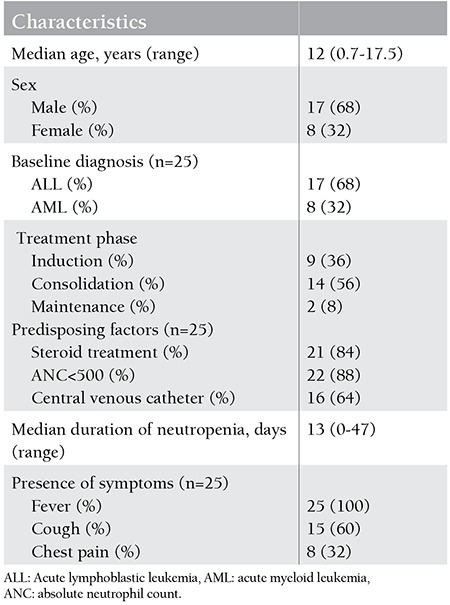
Characteristics of 25 children diagnosed with an invasive fungal infection.

**Table 2:**
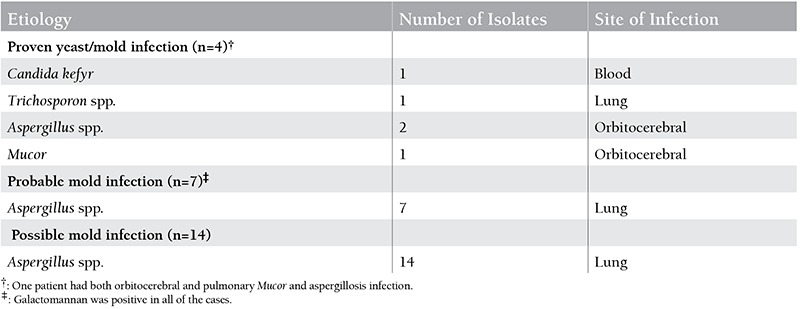
Classification, etiology, and sites of fungal infection.

**Table 3:**
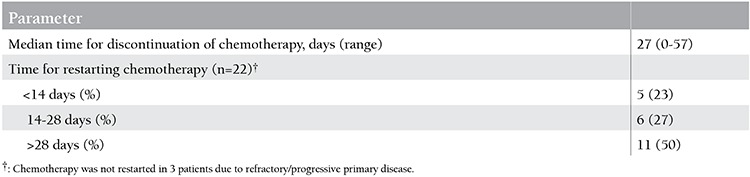
Time for discontinuation and restarting of chemotherapy in 22 patients.

**Table 4:**
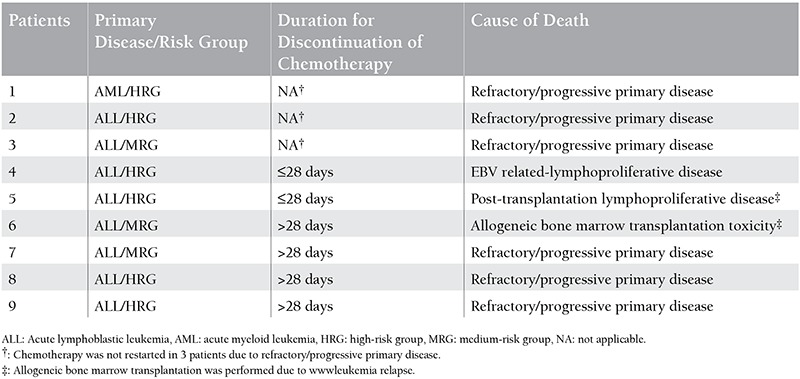
Disease characteristics, duration of chemotherapy discontinuation, and causes of death for the 9 patients who died.
